# Interaction between CETP Taq1B polymorphism and dietary patterns on lipid profile and severity of coronary arteries stenosis in patients under coronary angiography: a cross-sectional study

**DOI:** 10.1186/s12937-023-00899-w

**Published:** 2023-12-14

**Authors:** Azam AhmadiVasmehjani, SeyedMostafa SeyedHosseini, SayyedSaeid Khayyatzadeh, Farzan Madadizadeh, Mahta Mazaheri-Naeini, Mahdie Yavari, Zahra Darabi, Sara Beigrezaei, Marzieh Taftian, Vahid Arabi, Maryam Motallaei, Amin Salehi-Abargouei, Azadeh Nadjarzadeh

**Affiliations:** 1 Research Center for Food Hygiene and Safety, School of Public Health, Shahid Sadoughi University of Medical Sciences, Yazd, Iran; 2 Afshar Hospital Yazd Cardiovascular Research Center, Non-Communicable Diseases Research Institute, Shahid Sadoughi University of Medical Sciences, Yazd, Iran; 3 Department of Nutrition, School of Public Health, Shahid Sadoughi University of Medical Sciences, Yazd, Iran; 4 Center for healthcare Data modeling, Departments of biostatistics and Epidemiology, School of public health, Shahid Sadoughi University of Medical Sciences, Yazd, Iran; 5 Department of Medical Genetics, Faculty of Medicine, Shahid Sadoughi University of Medical Sciences, BP634, F8916978477, Yazd, Iran; 6 Mother and Newborn Health Research Center, Shahid Sadoughi Hospital, School of Medicine, Shahid Sadoughi University of Medical Sciences, Yazd, Iran; 7 Division of Genetics, Department of Cell and Molecular Biology and Microbiology, Faculty of Science and Biotechnology, University of Isfahan, Isfahan, Iran; 8 Dr. Mazaheri’s Medical Genetics Lab, Yazd, Iran

## Abstract

**Aim:**

Evidence indicates there are still conflicts regarding CETP Taq1B polymorphism and coronary artery disease risk factors. Current findings about whether dietary patterns can change the relationship of the Taq1B on lipid profile and the severity of coronary arteries stenosis appears to be limited. The present research made an attempt to investigate this possible relationship.

**Methods:**

This cross-sectional study involved 453 male and female participants with a mean age of 57 years. A validated 178-item food frequency questionnaire (FFQ) was used to assess dietary usual intake. Dietary patterns were extracted through principal component analysis (PCA). Taq1B variant was genotyped by the polymerase chain reaction-restriction fragment length polymorphism (PCR-RFLP) method. Two-way ANOVA was used to test the interaction between Taq1B polymorphism and dietary patterns.

**Results:**

Two dietary patterns were detected: the western dietary pattern (WDP) and the traditional dietary pattern (TDP). The frequency of Taq1B genotypes turned out to be 10.4, 72.4, and 17.2% for B1B1, B1B2, and B2B2, respectively. A significant difference was observed in TG and TG/HDL-C levels among TaqIB genotypes in higher adherence to TDP (*P* = 0.01 and *P* = 0.03, respectively). Taq1B showed a significant interaction with TDP for modulating TG levels and TG/HDL-C ratio (*P* = 0.02 and *P* = 0.04, respectively). Greater compliance to WDP demonstrated a significant difference in TG and TG/HDL-C levels across rs708272 genotypes (*P* = 0.03) after adjusting for confounding factors. Other lipid components and coronary arteries stenosis scores failed to show any relationship or significant difference across Taq1B genotypes or dietary patterns.

**Conclusion:**

Adherence to TDP may adjust the association between the Taq1B variant and TG and TG/HDL-C levels in patients undergoing coronary angiography. To better understand the relationships, we suggest prospective studies in different race groups with multivariate approaches.

**Supplementary Information:**

The online version contains supplementary material available at 10.1186/s12937-023-00899-w.

## Introduction

Coronary artery disease (CAD) is one of the major forms of cardiovascular disease (CVD) manifested with complications such as myocardial infarction (MI), angina stable/unstable, and sudden death [1]. The World Health Organization (WHO) states that CAD is the biggest contributor to disease burden worldwide and the main cause of mortality in developing countries [2, 3]. Risk factors, including age, gender, elevated blood pressure, decreased physical activity, smoking, unhealthy diet, abnormal levels of serum lipids including increased triglyceride (TG), low-density lipoprotein cholesterol (LDL-C), total cholesterol (TC) and decreased high-density lipoprotein cholesterol (HDL-C), unfavorable ratio in the lipoproteins, obesity, stress, and diabetes mellitus type 2 (DM2) have all been attributed to CAD upsurge, most of which can be modifiable [4, 5]. As reported by several studies [6, 7], one of the main risk factors for CAD is atherosclerosis. Atherosclerosis can be developed faster when plasma lipids are abnormal [8]. The evidence suggests that eating patterns can impact the risk of atherosclerosis by affecting plasma lipids [9, 10]. However, research is under way to understand the different responses that are observed among the individuals when they are exposed to a similar dietary pattern [11, 12]. Based on this, some investigations have proposed that genetic variations, especially single nucleotide polymorphisms (SNPs), may explain some of the diverse responses in populations [11].

The (rs708272) Taq1B SNP is located at the 277th nucleotide of the first intron of the cholesterol ester transfer protein (CETP) gene on the long arm of chromosome 16q-21 [13]. Taq1B variant is created by a silent mutation Guanine base (G) to Adenine base (A) so that G is called B1 allele (frequent allele) with a cutting site for Taq1 endonuclease enzyme and A is called B2 allele (less common allele) without Taq1 restriction site [14]. CETP gene encodes the plasma CETP which transfers cholesteryl esters from HDL-C to iatrogenic lipoproteins in exchange for TG [15].

A host of studies have investigated the association between the Taq1B variant and lipid profile and CAD risk, but the results have turned out to be inconsistent. Studies have explored that the B2 allele, compared to the B1 one, is associated with the risk of CAD and HDL-C levels through reduced CETP activity, [16, 17]; however, the findings may vary in different populations.

The Taq1B variant may interact with dietary components to influence lipid levels and CAD risk. There are conflicting reports on whether dietary fat intake can modify the effect of Taq1B polymorphism on plasma lipid levels [18–21]. Similarly, other studies have reported inconsistent results [15]. Dietary patterns account for the role of all dietary components and can reveal more real associations, but studies on the issue are very scarce.

We therefore made an effort to investigate whether the association of Taq1B with lipid profile levels and the severity of coronary artery stenosis is modified by adherence to dietary patterns in patients undergoing coronary angiography.

## Methods

This cross-sectional study included 453 patients aged 35 to 75 years of both genders. The sample was calculated through related parameters by Quanto soft ware (version 4.2.1). The participants had been referred to Afshar Hospital in Yazd, Iran, from September 2020 to October 2021 for coronary angiography. The exclusion criteria comprised a history of cancer, myocardial infarction (MI), chronic heart failure (CHF), percutaneous coronary intervention (PCI), coronary artery bypass grafting (CABG), kidney failure, liver disease and use of its medications, certain perceptual or psychological disorders, immune system failure, acquired immunodeficiency syndrome (AIDS), extreme obesity (body mass index (BMI) > 40 kg/m2), restriction in oral intake, pregnancy, and lactation. Prior to entering the subjects into the study, a written informed consent was obtained from them all. The current study was approved by the Ethical Committee of Shahid Sadoughi University (SSU) of Medical Sciences in Yazd, Iran (IR.SSU.SPH.REC.1400.079).

Genomic DNA was extracted from whole blood based on an extraction kit (Simbiolab, Iran). The CETP-TaqIB variant was genotyped by Polymerase Chain Reaction-Restriction.

Fragment Length Polymorphism (PCR-RFLP). A volume of the PCR consisted of 20-µL; 2 µL genomic DAN, 6 µL water, 10 µL Master Mix (Amplicon, Denmark), and 1 µL of each primer 5’-ACTAGCCCAGAGAGAGGAGTG-3’ as well as 5’-CAGCCGCACACTAACCCTA-3’ (Sina colon, Iran). The amplification protocol took account of 1 cycle of primary denaturation at 95 °C for 5 min, followed by 40 cycles (95 °C for 30 s, 66 °C for 30 s, 72 °C for 30 s) and, final extension at 72 °C for 5 min. Then PCR products were electrophoresed on 2% agarose gel (SinaClon, Iran) and were digested by endonuclease of the Taq1 (Fermentase, Lithuania). The digested solution contained 30 µL (10 µL PCR product, 2 µL buffer, 0.5 µL Taq1 enzymes, and 17.5 µL water) that was incubated at 37 °C overnight. Digested fragments of the 708,272-CETP were electrophoresed on 2% agarose gel with a voltage of 100 for 1 h.

After the overnight fasting, blood samples were drawn from the participants. Then serum was isolated through centrifuge of the samples (5000 rpm for 5 min, 4 °C). They were stored at -20 °C until analysis time. Lipid profiles such as triglyceride (TG), high-density lipoprotein cholesterol (HDL-C), Low-density lipoprotein cholesterol (LDL-C), and total cholesterol (TC) were measured using a commercial kit Pars Azmun (Tehran, Iran). Abnormal levels of lipid profile were later considered according to the National Cholesterol Education Program Adult Treatment Panel III (NCEP ATP III): TG ≥ 150 mg/dl, HDL-C < 40 mg/dl for males, and HDL-C < 50 mg/dl for females, LDL-C ≥ 130 mg/dl and TC ≥ 200 mg/dl [22].

Grade of coronary arteries stenosis was assessed by angiographic Gensini score (GS) as follows: 1 point for ≤ 25% obstruction, 2 points for 26–50%, 4 points for 51–75%, 8 points for 76–90%, 16 points for 91–99%, and 32 points for full occlusion. Depending on the type of coronary artery and obstructed sections, scores were multiplied by coefficients 1 to 5. A coefficient of 5 was considered for the left main coronary artery, 2.5 for the left anterior descending and proximal, 1.5 for the mid-segment of the left anterior descending coronary artery, 1 for the right proximal, and 0.5 for other segments. The final GS was the sum of stenosis scores and coefficients for each lumen [23, 24]. GS ≥ 20 was considered as intermediate-high-risk severity of coronary artery stenosis and that of < 20 as low-risk CAD [25].

Using an online calculator version 2.0, the SYNTAX score (SS) was calculated (http://syntaxscore.org/calculator). List questions SS regarded functional and anatomical parameters of the stenosis ≥ 50% in arteries with a diameter of ≥ 1.5 mm. Finally, scores was calculated to obtain sum of all obstruction scores. SS < 23 was considered as low severity of coronary artery stenosis and SS ≥ 23 as moderate-severe [26, 27]. The coronary angiographies were explained by experienced cardiologists who were blinded for all variables except age and sex.

Weight was measured using Omron BF-511 portable digital scales (with an accuracy of 100 gr) as well as height with a tape (with an accuracy of 0.1 cm). They were all measured according to standardized methods. Body mass index (BMI) was also calculated through the weight (kg) to square of height (m2) ratio. Waist circumference (WC) was measured through a non-stretch tape at the middle of the iliac crown and lowest rib in the standing position with the accuracy of 0.5 cm. Body fat percent (BFP) measurement was performed by Omron BF-511. All of these measurements were done by some trained nutritionists.

Daily physical activity was evaluated using the International Physical Activity Questionnaire (IPAQ) [28]. Activity levels were considered as metabolic equivalent (MET) hours per week and categorized as sedentary, moderate, or intense based on a list of usual activities per day over the previous week.

Usual dietary intake during the previous year was controlled using a 178-item FFQ which was a reliable and validated version of 168 food items [29, 30]. Intake frequency and amount of food items were demanded on a daily, weekly, monthly, and yearly basis. Portion sizes were converted to gram per day through household scales [31]. To extract consumed nutrients, Nutritionist 4 software (First Databank Inc. Hearst Corp, San Bruno, CA) was applied. To recognize dietary patterns, food items of the FFQ were categorized into 34 food groups based on their similarity in nutrient content and reported data obtained from some Iranian studies. Several food items were also classified separately. Data on dietary intake were assessed by trained nutritionists through face-to-face interviews.

Additional data including age, gender, smoking, job, educational levels as well as other socioeconomic and demographic data were collected via general questionnaires. Blood pressure was measured by hospital nurses before angiography and according to standard protocols.

To extract the dietary patterns of participants in the current study, we used factor analysis with principal component analysis (PCA) by orthogonal Varimax rotation. Factors were preserved by using scree plot, their explanation, and eigen values (EV) more than 1 [32]. Main factors were derived and named as to arbitrary decision and previous studies. Factor score for every dietary pattern for each subject was obtained from aggregating factor loadings (food groups weighted) [33]. Major dietary patterns were named through a rotated factor loading greater than 0.3 [34, 35]. Correlation among 34 food groups and adequacy sample size for factor analysis were determined by the Bartlett test and the Kaiser–Meyer–Olkin (KMO) test, respectively. Participants were then categorized to low and high adherents to dietary patterns based on a median of factor scores. One-way ANOVA was used to compare continuous variables across Taq1B genotypes while an independent sample t-test was used across low and high adherence to dietary patterns. Chi-square test was performed for categorical variables by Taq1B genotypes and adherence to dietary patterns. The differences in lipid profile and scores of the arteries stenosis in different adherents to dietary patterns among Taq1B genotypes were tested by One-way ANOVA and ANCOVA analysis in crude and adjusted models, respectively. However, Two-way ANOVA was deployed for interactions in both crude and adjusted models. Adjustments were made for age, gender, BMI, menstrual status, smoking status, medicine intake anti-diabetes, anti-lipid and anti-blood pressure, educational status and energy intake. The Pearson’s χ2 statistic was also applied for assessing the Hardy–Weinberg equilibrium (HWE). Statistical analyses were conducted using statistical package for the social sciences (SPSS) version 26.0 (IBM Corporation, USA). *P*-value < 0.05 was used as the significance level for all analyses.

## Results

According to the PCA method two dietary patterns were extracted: the first dietary pattern involved food groups with factor loading more than 0.3 such as; pickles, vegetables, fruits, sugar, red meats, natural fruit juice, broth, animal oils, nuts, butter, tea, and total dairy which were named the traditional dietary pattern (TDP). Nature of Iranian TDP is complex, it included both healthy and unhealthy foods as in this TDP have been loaded. Also unhealthy dietary components such as butter, animal fat, and red meat that are used in preparing traditional Iranian foods, have been loaded in this the TDP. The second dietary pattern was diagnosed by food groups with factor loading more than 0.3 such as; industrial Juices, sweet beverages, coffee, sweets desserts, egg, refined grains, vegetable oils, fish, processed meat, legumes, poultry, mayonnaises, and fried which stated as the western dietary pattern (WDP). These dietary patterns explained 16.9% of the overall variance in dietary intake. Data are shown in (Table 1**).**

**Table 1:** Food loading for extracted major dietary patterns

Food group	Traditional dietary pattern	Western dietary pattern
pickle	0.611	
Vegetables	0.579	
Fruits	0.533	
Sugar	0.514	
Red meats	0.456	
Fruit juice	0.441	
Broth	0.426	
Animal oils	0.385	
Nuts	0.383	
Butter	0.378	
Tea	0.365	
Total dairy	0.352	
Industrial Juices		0.543
Sweet beverages		0.535
Coffee		0.469
Sweets desserts		0.455
Eggs		0.455
Refined grains		0.449
Vegetable oils		0.399
Fish		0.389
Processed meat.		0.383
legumes		0.370
Poultry		0.360
Mayonnaises and fried		0.310
Variance explained (%)	8.83	8.08

Values less than 0.30 are not reported

General and dietary intake characteristics of participants in difference adherence to dietary patterns between CETP Taq1B genotypes are presented in (Table 2**).** Out of a total 453 participants consisted of 284 men (62.7%) and 169 women (37.3%). The participants had a mean standard deviation (SD) of age and BMI 56.85 (9.37) years and 27.42 (4.16) kg/m^2^, respectively. The frequency CETP Taq1B genotypes were observed 10.4%, 72.4%, and 17.2% for B1B1, B1B2, and B2B2, respectively. The frequency of minor allele A (B2) was 53.42%. Genotypes were within Hardy–Weinberg equilibrium (*P*-value > 0.999).

**Table 2:** Baseline characteristics and dietary intakes across CETP rs708272 genotypes and adherence of dietary patterns

Variables	CETP (rs708272)				WDP			TDP		
	B1B1	B1B2	B2B2	*P*-value^*^	Low	High	*P*-value^*^	Low	High	*P*-value^*^
**Number (percentage)**	47 (10.4)	328(72.4)	78 (17.2)		226	227		226	227	
**Gender**				0.96			< 0.001			< 0.001
**Male, n (%)**	30(63.8)	206(62.8)	48(61.5)		109(48.2)	175(77.1)		116(51.3)	168(74)	
**Female, n (%)**	17(36.2)	122(37.2)	30(38.5)		117(51.8)	52(22.9)		110(48.7)	59(26)	
**Age, year**	57.77 ± 10.37	56.77 ± 9.14	56.66 ± 9.80	0.77	58.75 ± 8.64	54.97 ± 9.70	< 0.001	56.62 ± 9.46	57.08 ± 9.29	0.60
**WC,cm**	100.58 ± 11.34	99.30 ± 12.52	100.32 ± 9.80	0.67	100.78 ± 11.88	98.43 ± 11.96	0.036	100.70 ± 12.08	98.51 ± 11.77	0.052
**BMI, kg/m** ^**2**^	27.27 ± 4.06	27.30 ± 4.21	28.00 ± 4.01	0.39	27.83 ± 4.26	27.01 ± 4.02	0.036	28.00 ± 4.34	26.84 ± 3.89	0.003
**BFP**	31.58 ± 10.56	31.70 ± 10.66	32.87 ± 10.16	0.66	34.14 ± 10.76	29.64 ± 9.86	< 0.001	33.87 ± 11.03	29.91 ± 9.67	< 0.001
**Physical activity, n (%)**				0.83			0.15			0.073
**Sedentary**	16(34)	111(34.4)	25(32.1)		85(37.9)	67(29.9)		88(38.9)	64(28.8)	
**Moderate**	18(38.3)	105(32.5)	24(30.8)		72(32.1)	75(33.5)		70(31)	77(34.7)	
**Active**	13(27.7)	107(33.1)	29(37.2)		67(29.9)	82(36.6)		68(30.1)	81(36.5)	
**Education, n (%)**				0.15			< 0.001			0.16
**Uneducated**	5(10.9)	74 (22.6)	18(23.1)		68(30.2)	29(12.8)		55(24.4)	42(18.6)	
**Unacademic**	34(73.9)	231(70.6)	53(67.9)		141(62.7)	177(78.3)		156(69.3)	162(71.7)	
**Academic**	7 (15.2)	22(6.7)	7(9)		16(7.1)	20(8.8)		14(6.2)	22(9.7)	
**Menopausal status, yes, n(%)**				0.91			< 0.001			< 0.001
	3(6.4)	30(9.1)	9(11.5)		25(11.1)	17(7.5)		28(12.4)	14(6.2)	
**Smoking status, n (%)**				0.72			< 0.001			< 0.001
Non smoker	29(61.7)	211(64.3)	54(69.2)		173(76.5)	121(53.3)		169(74.8)	125(55.1)	
Former smoker	2(4.3)	14(4.3)	1(1.3)		7(3.1)	10(4.4)		8(3.5)	9(4)	
Current smoker	16(34)	103(31.4)	23(29.5)		46(20.4)	96(42.3)		49(21.7)	93(41)	
Medicine consumption, yes n(%)										
**Anti-hypertensives**	24(51.1)	144(43.9)	34(43.6)	0.64	122(54)	80(35.2)	< 0.001	105(46.5)	97(42.7)	0.42
**Anti-hyperlipidemic**	15(31.9)	118(36)	30 (38.5)	0.76	98(43.4)	65(28.6)	0.001	94(41.6)	69(30.4)	0.01
**Anti-diabetic**	15(31.9)	109(33.2)	23 (29.5)	0.81	85(37.6)	62(27.3)	0.01	87(38.5)	60(26.4)	0.006
**Nutrient intake**										
**Total energy (kcal/day)**	2604.86 ± 1188.33	2745.53 ± 1269.89	2553.57 ± 945.09	0.39	2026.63 ± 783.96	3366.17 ± 1194.27	< 0.001	2161.44 ± 1030.73	3231.96 ± 1142.51	< 0.001
**Carbohydrates, gr/d**	384.64 ± 194.83	421.25 ± 200.84	386.41 ± 147.10	0.21	317.44 ± 134.21	505.05 ± 195.95	< 0.001	330.81 ± 168.86	491.73 ± 180.56	< 0.001
**Proteins, gr/d**	100.20 ± 52.60	103.16 ± 54.97	96.57 ± 42.75	0.60	72.52 ± 32.67	130.78 ± 52.98	< 0.001	84.58 ± 48.67	118.77 ± 51.26	< 0.001
**Fats, gr/d**	78.48 ± 38.32	76.60 ± 44.53	75.49 ± 37.17	0.93	56.40 ± 29.39	96.72 ± 44.34	< 0.001	57.77 ± 30.01	95.36 ± 45.12	< 0.001
**MUFA, gr/d**	23.73 ± 12.33	23.22 ± 13.14	22.40 ± 10.50	0.82	16.78 ± 8.58	29.45 ± 12.84	< 0.001	17.94 ± 9.26	28.29 ± 13.40	< 0.001
**PUFA, gr/d**	18.75 ± 11.05	18.73 ± 13.55	17.71 ± 10.36	0.81	12.63 ± 7.47	24.45 ± 14.21	< 0.001	13.93 ± 8.45	23.16 ± 14.62	< 0.001
**SFA, gr/d**	21.98 ± 10.36	23.40 ± 15.21	21.87 ± 10.76	0.60	18.15 ± 12.37	27.81 ± 14.06	< 0.001	16.92 ± 9.01	29.03 ± 15.57	< 0.001
**Dietary fiber, gr/d**	36.97 ± 20.97	40.19 ± 22.40	36.33 ± 18.11	0.27	31.04 ± 17.35	47.30 ± 22.37	< 0.001	29.12 ± 15.11	49.22 ± 22.42	< 0.001
**Cholesterol, gr/d**	465.38 ± 297.73	445.69 ± 275.13	432.12 ± 232.80	0.80	336.34 ± 206.16	553.97 ± 283.06	< 0.001	377.38 ± 216.36	513.11 ± 300.56	< 0.001

BMI, body mass index; WC, waist circumference; BFP, body fat percentage; MUFA, mono unsaturated fatty acid; PUFA, poly unsaturated fatty acid; SFA, saturated fatty acid; WDP, western dietary pattern; TDP, traditional dietary pattern

Adherence to dietary patterns is **considered** two categories, “high” adherence and “low” adherence, according the median intake

Values are presented means ± standard deviation (SDs), n (%): numbers (percentage). ^*^Obtained from one- way ANOVA, independent t-test for continuous variables and Chi–square test for categorical. *P*-Values < 0.05 were considered significant

Comparing the highest adherence to TDP versus the lowest, participants tended to have lower BMI, and BFP (*P* = 0.003, *P* < 0.001, respectively). Subjects with higher adherence to both WDP and TDP had significantly a lower intake of medicines (anti-diabetes, anti-lipid) and also a lower number of them were in status of menopause (*P* < 0.05). More percentage of subjects who had adhered more to both WDP and TDP were current smokers (*P* < 0.001). Intake of nutrients including energy, protein, carbohydrate, total fat, monounsaturated fatty acid (MUFA), saturated fatty acid (SFA), polyunsaturated fatty acid (PUFA), cholesterol, and fiber in participants with the highest adherence to WDP and TDP significantly were higher compared with those with the lowest adherence (*P* < 0.001). No significant difference was observed for other general and dietary variables by CETP Taq1B genotypes.

Using two-way ANOVA revealed a significant interaction between CETP Taq1B polymorphism with adherence to TDP on levels of TG and on TG/HDL-C ratio in both crude and adjusted models (*P* = 0.02 and *P* = 0.04, respectively), it observed after controlling confounders for gender, smoking status, menstrual status, BMI, energy, medications (anti-hyperlipidemia and anti-diabetic). data presented in Table 3. No interaction was found between Taq1B variant and adherence to TDP on other lipid profile and scores of arteries stenosis. In higher adherence to TDP, significant distribution of TG levels observed across Taq1B genotypes, in both crude and adjusted models (*P* = 0.03 and *P* = 0.01, respectively). TG/HDL-C ratio was significantltly different among Taq1B genotypes in greater compliance to TDP, it observed after adjusting for confounders (*P* = 0.03).Table 3.

**Table 3:** Interaction between the CETP TaqIB polymorphism and adherence to TDP on CVD risk factors

Variables	Model Crude			*P* ^*a*^	P**	Model adjusted			*P* ^*b*^	P**
	CETP Taq1B genotype					CETP Taq1B genotype				
	B1B1 n = 47	B1B2 n = 328	B2B2 n = 78			B1B1 n = 47	B1B2 n = 328	B2B2 n = 78		
	mean ± SE	mean ± SE	mean ± SE			mean ± SE	mean ± SE	mean ± SE		
HDL-c (mg/d)										
**Low**	47.15 ± 2.42	49.71 ± 0.97	52.19 ± 1.88	0.30	0.72	47.59 ± 2.57	49.65 ± 0.92	52.21 ± 2.0	0.33	0.64
**High**	46.66 ± 2.13	47.11 ± 0.81	48.25 ± 1.92	0.79		46.92 ± 2.15	47.16 ± 0.85	47.88 ± 1.67	0.91	
LDL-c (mg/dl)										
**Low**	92.42 ± 9.18	102.20 ± 3.32	105.67 ± 7.17	0.51	0.79	95.72 ± 8.97	101.81 ± 3.22	105.46 ± 6.99	0.69	0.78
**High**	87.42 ± 7.37	94.63 ± 2.91	92.03 ± 5.68	0.64		87.16 ± 7.49	94.56 ± 2.95	92.45 ± 5.81	0.64	
TC(mg/dl)										
**Low**	185.20 ± 26.23	211.39 ± 8.24	215.09 ± 21.96	0.56	0.23	196.22 ± 23.61	210.66 ± 8.48	211.75 ± 18.40	0.84	0.39
**High**	214.63 ± 28.54	189.06 ± 7.71	181.41 ± 11.32	0.38		215.06 ± 19.55	187.47 ± 7.71	183.39 ± 15.16	0.38	
**TG(mg/dl)**										
**Low**	136.56 ± 12.74	151.52 ± 5.52	153.02 ± 15.05	0.65	0.04	139.40 ± 14.86	151.78 ± 5.34	150.09 ± 11.58	0.73	0.02
**High**	198.40 ± 23.80	151.90 ± 5.84	173.84 ± 18.77	0.03		198.18 ± 17.34	150.79 ± 6.84	178.21 ± 13.44	0.01	
**TG/HDL**										
**Low**	3.02 ± 0.32	3.15 ± 0.12	2.90 ± 0.22	0.67	0.10	3.06 ± 0.32	3.16 ± 0.11	2.84 ± 0.25	0.51	0.04
**High**	4.30 ± 0.38	3.33 ± 0.13	3.60 ± 0.32	0.06		4.28 ± 0.36	3.30 ± 0.14	3.75 ± 0.28	0.03	
Gensisni score										
**Low**	25.81 ± 8.76	32.37 ± 2.91	25.56 ± 5.27	0.49	0.84	28.32 ± 7.54	32.11 ± 2.71	25.27 ± 5.88	0.54	0.76
**High**	34.49 ± 8.67	38.86 ± 3.32	37.91 ± 7.90	0.89		31.12 ± 8.63	39.32 ± 3.40	38.16 ± 6.69	0.67	
Syntax score										
**Low**	8.00 ± 3.10	10.10 ± 0.91	8.99 ± 2.13	0.70	0.93	8.91 ± 2.49	9.97 ± 0.89	9.03 ± 1.94	0.85	0.88
**High**	10.22 ± 2.63	11.53 ± 0.97	11.54 ± 2.24	0.89		9.54 ± 2.55	11.56 ± 1.00	11.83 ± 1.97	0.73	

TDP, traditional dietary pattern; BMI, body mass index; TC; total cholesterol, HDL-C; high-density lipoprotein cholesterol, LDL-C; low-density lipoprotein cholesterol, TG; triglyceride. Adherence to dietary patterns is **considered** two categories, “high” adherence and “low” adherence, according the median intake

Values are presented means ± standard errors (SEs), ^a^ P based on One- way ANOVA, ^b^ P adjusted for gender, smoking status, menopausal status, BMI, energy, medications. P** obtained from Two- way ANOVA both model crude and adjusted

*P*-Values < 0.05 were considered significant

No interaction was found between Taq1B variant with adherence to WDP on lipid profile, GS and SS. We observed in greater adherence to WDP, TG and TG/HDL-C levels were significantly distributed across Taq1B genotypes (*P* = 0.03), after adjusting for (age, gender, BMI, menstrual status, smoking status, medicine intake anti-diabetes, anti- hyperlipidemia and anti-hypertensive, educational status and intake of energy). Table 4.

**Table 4:** Interaction between the CETP TaqIB polymorphism and adherence to WDP on CVD risk factors

Variables	Model Crude			*P* ^*a*^	P**	Model adjusted			*P* ^*b*^	P**
	CETP Taq1B genotype					CETP Taq1B genotype				
	B1B1 n = 47	B1B2 n = 328	B2B2 n = 78			B1B1 n = 47	B1B2 n = 328	B2B2 n = 78		
	mean ± SE	mean ± SE	mean ± SE			mean ± SE	mean ± SE	mean ± SE		
HDL-c (mg/d)										
**Low**	50.41 ± 3.09	49.29 ± 0.88	50.22 ± 2.26	0.86	0.42	49.83 ± 2.75	49.08 ± 0.91	51.22 ± 1.94	0.60	0.65
**High**	44.50 ± 1.53	47.53 ± 0.92	49.92 ± 1.60	0.13		45.52 ± 2.16	47.59 ± 0.87	49.16 ± 1.78	0.43	
LDL-c (mg/dl)										
**Low**	102.25 ± 8.64	96.48 ± 2.82	93.35 ± 6.23	0.69	0.09	103.62 ± 8.57	95.89 ± 2.85	93.96 ± 6.06	0.64	0.11
**High**	81.29 ± 5.71	100.66 ± 3.23	103.04 ± 8.89	0.07		83.02 ± 8.48	101.37 ± 3.44	99.37 ± 6.99	0.13	
TC(mg/dl)										
**Low**	188.65 ± 22.92	195.08 ± 8.40	202.91 ± 19.99	0.88	0.65	193.25 ± 25.34	193.95 ± 8.43	206.58 ± 17.91	0.81	0.26
**High**	209.14 ± 28.92	205.25 ± 7.61	191.29 ± 13.53	0.70		213.11 ± 19.11	207.87 ± 7.76	180.11 ± 15.76	0.25	
TG(mg/dl)										
**Low**	163.39 ± 23.40	161.79 ± 5.93	168.97 ± 18.98	0.90	0.46	170.77 ± 19.01	159.18 ± 6.32	175.24 ± 13.44	0.51	0.45
**High**	173.57 ± 18.95	140.99 ± 5.24	159.73 ± 15.86	0.07		176.76 ± 14.52	140.67 ± 5.89	161.50 ± 11.97	0.03	
TG/HDL										
**Low**	3.30 ± 0.43	3.38 ± 0.13	3.36 ± 0.31	0.97	0.22	3.45 ± 0.38	3.34 ± 0.12	3.45 ± 0.27	0.91	0.20
**High**	3.98 ± 0.47	3.09 ± 0.12	3.20 ± 0.27	0.05		4.01 ± 0.33	3.07 ± 0.13	3.28 ± 0.27	0.03	
Gensisni score										
**Low**	35.43 ± 9.90	36.95 ± 3.16	25.84 ± 5.27	0.30	0.26	38.49 ± 8.75	36.20 ± 2.91	28.51 ± 6.18	0.49	0.39
**High**	27.03 ± 7.90	34.03 ± 3.07	38.26 ± 8.14	0.54		25.01 ± 7.78	35.01 ± 3.16	36.39 ± 6.41	0.45	
Syntax score										
**Low**	9.95 ± 2.83	11.17 ± 0.96	8.27 ± 1.80	0.41	0.29	10.67 ± 2.72	11.01 ± 0.90	8.90 ± 1.92	0.61	0.45
**High**	8.66 ± 2.80	10.40 ± 0.92	12.35 ± 2.48	0.49		8.27 ± 2.41	10.68 ± 0.98	11.72 ± 1.98	0.53	

WDP, western dietary pattern; BMI, body mass index; **TC;** total cholesterol, HDL-C; high-density lipoprotein cholesterol, LDL-C; **low**-density lipoprotein cholesterol, **TG;** triglyceride. Adherence to dietary patterns is considered two categories, “high” adherence and “low” adherence, according the median intake

Values are presented means ± standard errors (SEs), ^a^ P based on One- way ANOVA, ^b^ P adjusted for gender, smoking status, menopausal status, BMI, energy, medications, education, age. P** obtained from Two- way ANOVA *both* model crude and adjusted

*P*-Values < 0.05 were considered significant

**Fig. 1:**
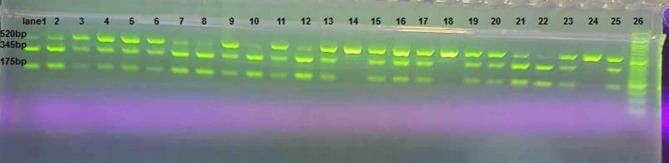
This figure shows the digested fragments of the 708,272-CETP on 2% agarose gel electrophoresis. The ladder marker (lane 26) was 50 bp, The heterozygous B1B2 genotype had three bands of 175 bp, 345 and 520 bp (lane 1–13, 15–17, 19–21, 23 and 25) and The homozygous B2B2 genotype had one band of 520 bp (lane 14, 18 and 24) as well as The homozygous B1B1 genotype had two bands 175 bp 345 bp (lane 22)

Analysis of digested products on 2% agarose gel contained in three fragments: uncut homozygous B2B2 that had one band (520 bp (bp), cut heterozygous B1B2 with three bands (175, 345, and 520 bp) and cut homozygous B1B1 with two bands (175, 345 bp). Figure 1.

## Results

### Study population characteristics

Out of 453 participants included 284 men (62.7%) and 169 women (37.3%). The mean standard deviation (SD) of age and BMI of the participants were 56.85 (9.37) years and 27.42 (4.16) kg/m^2^, respectively. The CETP Taq1B genotypes were distributed as follows: 10.4% for B1B1, 72.4% for B1B2, and 17.2% for B2B2. The minor allele A (B2) had a frequency of 53.42%. Genotypes were within Hardy–Weinberg equilibrium (*P*-value > 0.999).

Among the study population, two dietary patterns were identified by the PCA method: the first was characterized by high consumption of pickles, vegetables, fruits, sugar, red meats, natural fruit juice, broth, animal oils, nuts, butter, tea, and total dairy which were named as the traditional dietary pattern (TDP). Nature of Iranian TDP is complex, it included both healthy and unhealthy foods as in this TDP have been loaded. Also unhealthy dietary components such as butter, animal fat, and red meat that are used in preparing traditional Iranian foods as have been loaded in this the TDP. The second was identified by high intake food groups such as industrial Juices, sweet beverages, coffee, sweets desserts, egg, refined grains, vegetable oils, fish, processed meat, legumes, poultry, mayonnaises, and fried which stated as the western dietary pattern (WDP). These dietary patterns explained 16.9% of the overall variance in dietary intake. Data are shown in (Table 1).

The general and dietary intake characteristics of the participants according to their adherence to the dietary patterns and their CETP Taq1B genotypes are shown in Table 2. Participants who had the highest adherence to TDP had lower BMI and BFP (*P* = 0.003, *P* < 0.001, respectively) compared to those who had the lowest adherence. Subjects with higher adherence to both WDP and TDP had significantly a lower use of medicines (anti-diabetes, anti-lipid) additionally, fewer of them were in menopausal status (*P* < 0.05). A higher percentage of subjects who adhered more to both WDP and TDP were current smokers (*P* < 0.001). Intake of nutrients including energy, protein, carbohydrate, total fat, monounsaturated fatty acid (MUFA), saturated fatty acid (SFA), polyunsaturated fatty acid (PUFA), cholesterol, and fiber in participants with the highest adherence to WDP and TDP significantly were higher compared with those with the lowest adherence (*P* < 0.001). No significant difference was observed in the general and dietary variables across CETP Taq1B genotypes.

### Comparison of lipid profile and scores of coronary arteries stenosis in adherence to dietary patterns among Taq1B genotypes

In higher adherence to TDP, significant distribution in TG levels observed across Taq1B genotypes, in both crude and adjusted models (*P* = 0.03 and *P* = 0.01, respectively). Additionally, the TG/HDL-C ratio was significantltly different among the Taq1B genotypes in greater compliance to TDP, in the adjusted model (*P* = 0.03). The confounders that were adjusted included gender, smoking status, menopausal status, BMI, energy intake, use of anti-diabetes, anti- hyperlipidemia medications, as presented in Table 3. In higher adherence to WDP, there was a significant difference in TG and TG/HDL-C levels among the Taq1B genotypes (*P* = 0.03), after adjusting for age, gender, BMI, menstrual status, smoking status, medication use (anti-diabetes, anti- hyperlipidemia and anti-hypertensive), educational level and energy intake, as shown in Table 4. There was no significant difference in the distribution of the other lipid parameters and the scores of coronary arteries stenosis among the Taq1B genotypes according to the adherence to TDP and WDP, as indicated in Tables 3 and 4.

### Interaction between the Taq1B polymorphism and dietary patterns on lipid profile and scores of coronary arteries stenosis

Using two-way ANOVA revealed a significant interaction between Taq1B variant with adherence to TDP on levels of TG in both crude and adjusted models (*P* = 0.04 and *P* = 0.02, respectively); and also on the TG/HDL-C ratio in the adjusted model (*P* = 0.04). Gender, smoking status, menstrual status, BMI, energy intake, medication use (anti-hyperlipidemia and anti-diabetic) controlled as confounding factors. No interaction was found between Taq1B variant and adherence to TDP on the other lipid parameters and the scores of coronary arteries stenosis, as presented in Table 3. No interaction was found between Taq1B variant with adherence to WDP on lipid profile and scores of coronary arteries stenosis, Data presented in Table 4.

Analysis of digested products on 2% agarose gel contained in three fragments: uncut homozygous B2B2 that had one band (520 bp (bp), cut heterozygous B1B2 with three bands (175, 345, and 520 bp) and cut homozygous B1B1 with two bands (175, 345 bp), as shown in Fig. 1.

## Discussion

This cross-sectional study investigated the interaction between Taq1B polymorphism and two dietary patterns (TDP and WDP) on the lipid profile and the severity of coronary arteries stenosis. Although there are many investigations regarding the Taq1B polymorphism and plasma lipid levels, to our knowledge, this is the first study to examine the association of SNP in combination with dietary patterns on plasma lipid levels and the severity of coronary arteries stenosis.

Our findings revealed the frequency of the B2 allele being greater than that of B1 (53% vs. 47%) thus being in line with some studies in Asia [36, 37] but contrary to others [38–41]. The results may vary depending on different ethnic groups and subjects’ clinical conditions. We found a significant difference in TG levels and TG/HDL-C ratio among Taq1B variant genotypes in the group with higher adherence to TDP. Moreover, Taq1B variant interacted with adherence to TDP in influencing TG levels and TG/HDL-C ratio.

Studies have identified that Taq1B2 variant is associated with higher HDL-C levels, lower TG and LDL-C levels and reduced stenosis of the arteries through reduced CETP concentration and activity [42–44]. Being questionable, however, these issues are yet to be resolved [38, 41, 45]. Based on the evidence, our results suggest that the B2 allele may have a protective role in lipid metabolism by influencing some lipid parameters. However, genetic studies have revealed that genes can only account for about 50% of the variation in plasma lipid levels [40]. Based on Corella’s et al. report, Taq1B variant can induce 5.8% of the HDL-C variance in a Spanish population [46]. It suggests that diet and dietary components may affect plasma lipid levels and vessel stenosis [40, 47]. However, evidence in this regard is also conflicting [48–50].

In this study, TDP consisted of high-fat foods along with healthy ones, including vegetables, fruits, nuts, and natural fruit juice. Consistent with our findings, some large scale studies have displayed no significant interaction between Taq1B variant and high intake of dietary fat on serum lipid profile [18, 51]. However, Kalantar et al. [52]. reported an interaction between a high-fat diet (> 34.9% of total energy intake) and Taq1B on TG/HDL-C ratio and HDL, which was more evident in those with the B1B1 genotype. Moreover, Li et al. [53] identified that in diabetic males without dyslipidemia, rs708272 B2 allele carriers bear higher HDL-C levels than those with the B1B1 genotype but indicate no effect on other lipid levels [53, 54]. These differences in the mentioned studies could be due to sample size and health status of their participants. This could also be ascribed to the effects of both unhealthy and healthy foods in the Iranian TDP. Consistent with this finding, Mirmiran et al. [10] explained that the Iranian TDP contains a mixed nature of both healthy and unhealthy food items, which may modify the combined effect of foods on genetic variants. Cosonant with this explanation, Serafini et al. also demonstrated that consuming two different foods can neutralize or diminish the antioxidant effect of the other [55]. Besides having high-fat items, the TDP in this study was also rich in nutrients comprising fibers, bioactive compounds, and phytochemicals from fruits, vegetables, and natural fruit juice. These nutrients may interact with Taq1B variant and affect TG metabolism and gene expression related to the β-oxidation of fatty acids [54]. In addition, prospective epidemiological studies have uncovered an inverse relationship between HDL-C levels and the risk of CAD [56]. A review study on this topic reported that the TaqIB variant genotypes of the CETP gene are strongly associated with HDL-C levels and the risk of CAD [17]. However, we failed to find such association in our study, thus being consistent with some recent investigations [39, 57]. Possible explanation Taq1B varian heteroginity, sample size, type of study design and Interaction of SNPs with each other. Further research is, therefore, needed to elucidate the exact mechanisms of Taq1B variant and TDP on lipid metabolism and coronary artery stenosis.

We also did not detect any relationship between WDP and Taq1B in modulating plasma lipid levels and the severity of coronary arteries. However, in the group with high adherence to WDP, TG level was significantly different across variant genotypes. In this study, the WDP was characterized by a high load of refined carbohydrates, such as industrial juices, sweet beverages, and sweets desserts and refined grains. Campos-Perez et al. in their study [58] demonstrated that sucrose intake over 5% of total energy intake in interacted with carriers of the B2 allele compared with B1B1 genotype bears higher TC and LDL-C levels. However, this finding is not conclusive due to the small sample size and the influence of environmental factors. Furthermore, Esmailzadeh et al. [59] reported that refined grain intake can be a contributory factor for high TG and low HDL-C levels which are the most common types of dyslipidemia in the Iranian population likely having the capacity to impair TG metabolism. Kokkou et al. [60] also reported that a WDP with higher intake of fat-rich foods, red meat and carbohydrate can be associated with greater severity of artery stenosis. In addition, Mohammad shahi et al. [50] unvovered a positive association between the greater compliance of WDP and the severity of coronary artery stenosis.

High-fat foods in the WDP of this study, including red meat, mayonnaise and fried foods, are some important contributory factors for CAD, as a cohort study conducted by Mirmiran et al. [10] also indicated that high adherence to the WDP could be related to the risk of CVD events. Further, Kuhail et al. [61] demonstrated that a diet high in refined carbohydrates is directly related to severity of artery stenosis based on GS. This is consistant with what Bhupathiraju et al. [62] explained that the problem could be due to the increased appetite, insulin dysfunction and calorie intake from refined carbohydrates. In this study, the lower TG levels in B1B2 carriers with higher adherence to WDP compared to the B1B1 genotype could be attributed to the protective role of the B2 allele in reducing CETP activity and TG levels, as reported by some previous studies [43, 44].

This study had several strengths, such as adjusting for many potential confounders in the analysis and using a reliable and validated questionnaire to measure dietary intake. The probe, however, had some limitations one of which was the cross-sectional design thus not allowing us to establish causality. Furthermore, we did not measure plasma CETP or HDL3 levels, which are related to plasma CETP levels [63].

## Conclusion

Adherence to TDP can significantly affect the association between Taq1B variant and TG and TG/HDL-C levels in patients undergoing coronary angiography. However, WDP proved to have no effect on the lipid profile in interaction with the TaqIB variant. To validate our findings as for gene-diet interaction on lipid profile, future studies are needed to be carried out in other ethnicities or in general populations.

### Electronic supplementary material

Below is the link to the electronic supplementary material.

Supplementary Material 1

## Data Availability

The data and materials of the current study are available from the corresponding author on reasonable request.
