# Supplementary figures and images for: Interaction between CETP Taq1B polymorphism and dietary patterns on lipid profile and severity of coronary arteries stenosis in patients under coronary angiography: a cross-sectional study
Source: Nutr J. 2023 Dec 14;22:70. doi: 10.1186/s12937-023-00899-w (PMC10720056; doi:10.1186/s12937-023-00899-w)

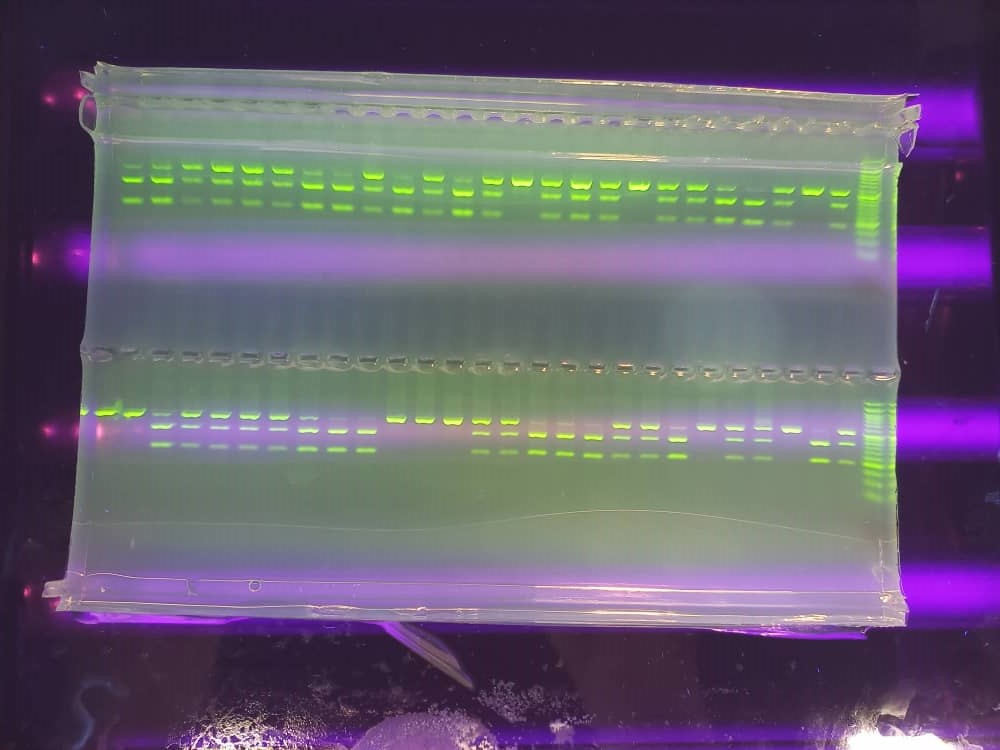

Supplement: Supplementary file 1 — Supplementary Material 1 [file 12937_2023_899_MOESM1_ESM.jpeg]
